# Mechanisms of Antibiotic and Biocide Resistance That Contribute to *Pseudomonas aeruginosa* Persistence in the Hospital Environment

**DOI:** 10.3390/biomedicines11041221

**Published:** 2023-04-19

**Authors:** Cláudia Verdial, Isa Serrano, Luís Tavares, Solange Gil, Manuela Oliveira

**Affiliations:** 1 Gato Escondido—Veterinary Clinic, Av. Bombeiros Voluntários n°22B, 2950-209 Palmela, Portugal; cssverdial@gmail.com; 2 CIISA—Center for Interdisciplinary Research in Animal Health, Faculty of Medicine, University of Lisbon, Avenida da Universidade Técnica, 1300-477 Lisboa, Portugal; ltavares@fmv.ulisboa.pt (L.T.); solange@fmv.ulisboa.pt (S.G.); moliveira@fmv.ulisboa.pt (M.O.); 3 Associate Laboratory for Animal and Veterinary Sciences (AL4AnimalS), 1300-477 Lisboa, Portugal

**Keywords:** antibiotic resistance, biocide tolerance, hospital environment, inert materials, *Pseudomonas aeruginosa*, virulence factors

## Abstract

*Pseudomonas aeruginosa* is an opportunistic bacterial pathogen responsible for multiple hospital- and community-acquired infections, both in human and veterinary medicine. *P. aeruginosa* persistence in clinical settings is worrisome and is a result of its remarkable flexibility and adaptability. This species exhibits several characteristics that allow it to thrive under different environmental conditions, including the ability to colonize inert materials such as medical equipment and hospital surfaces. *P. aeruginosa* presents several intrinsic mechanisms of defense that allow it to survive external aggressions, but it is also able to develop strategies and evolve into multiple phenotypes to persevere, which include antimicrobial-tolerant strains, persister cells, and biofilms. Currently, these emergent pathogenic strains are a worldwide problem and a major concern. Biocides are frequently used as a complementary/combination strategy to control the dissemination of *P. aeruginosa*-resistant strains; however, tolerance to commonly used biocides has also already been reported, representing an impediment to the effective elimination of this important pathogen from clinical settings. This review focuses on the characteristics of *P. aeruginosa* responsible for its persistence in hospital environments, including those associated with its antibiotic and biocide resistance ability.

## 1. Introduction

*Pseudomonas aeruginosa*, an aerobic, Gram-negative, rod-shaped bacterium, exhibits several characteristics that allow it to thrive under different environmental conditions. It is a ubiquitous microorganism that can be found both in abiotic and biotic environments, being able to tolerate a wide range of temperatures, varying from 4 °C to 42 °C [1,2]. Soil and water environments are common habitats for this bacterial species, whose opportunistic nature can partially explain its association with several hospital- and community-acquired infections [1,3,4,5,6].

*P. aeruginosa* is associated with high morbidity and mortality rates, both in human and veterinary medicine, especially in immunocompromised individuals [3,7]. In fact, according to the Centers for Disease Control and Prevention, in 2017 *P. aeruginosa* caused approximately 32,000 infections among hospitalized patients, being responsible for 2700 deaths in the United States alone [8]. This bacterium has been associated with several types of hospital-acquired infections, including ventilator-associated pneumonia, urinary tract infections in patients with long-term urinary catheterization, wound infections, bloodstream infections, and otitis [5,6,7,8,9,10,11,12]. *P. aeruginosa* infections can range from mild to life-threatening conditions, associated with treatment failure [1,5]. This species is also a major threat in veterinary medicine, being associated with chronic otitis externa, pyoderma, conjunctivitis, septicemia, lower urinary tract infections, pneumonia, and bacterial endocarditis [13,14,15,16,17,18,19,20,21,22,23,24,25].

The pathogenic potential of *P. aeruginosa* is related to its ability to express several virulence factors, including survival and stress-resistant mechanisms. In fact, this bacterial species frequently presents a multidrug-resistant profile and biofilm production ability, traits that render its elimination challenging. *P. aeruginosa*’s resistance to several antibiotics, including aminoglycosides, quinolones, and β-lactams, has been reported, and the emergence of new carbapenemase-producing strains is of major concern [23,26]. In fact, *P. aeruginosa* carbapenem-resistant strains have been classified by the World Health Organization since 2017 as critical-priority pathogens for the development of new antibiotics [27].

*P. aeruginosa*’s capacity to survive in different environments is also worrisome, as it can grow on a wide variety of surfaces, ranging from medical equipment and devices to sinks and drains from water systems [9,11,28,29,30,31,32]. The development of biofilms and persister cells poses a major challenge for surface disinfection and demands the characterization of the efficacy of disinfectants against these antimicrobial-resistant and -tolerant phenotypes [33,34,35,36,37,38].

This article reviews *P. aeruginosa*’s characteristics associated with its survival and persistence in hospital environments and surfaces, which represent a major concern in terms of nosocomial infections.

## 2. Genomic Structure

*P. aeruginosa* has the remarkable ability to adapt to a wide range of adverse and stressful environmental conditions. Having a large genome, of approximately 6.3 million base pairs in size and containing over 6000 protein-coding genes, this bacterial species presents a high genomic plasticity [39,40,41]. Its genetic material is divided into the core genome (~4 Mb) and the accessory genome, which mainly consists of variable-length and non-conserved extrachromosomal elements, such as plasmids. These mobile elements may include genomic islands [42], which are highly responsible for the versatility of this pathogen as they may contain genes related to virulence and antibiotic resistance abilities [40,42].

*P. aeruginosa*’s genome contains several genes involved in metabolic pathways, transport systems, and motility. The determinants responsible for the intrinsic resistance profile of this pathogen (i.e., associated with efflux pumps and antibiotic-inactivating enzymes) can be found in the core genome [39,41,42] and are also associated with its metabolic versatility and adaptability capacity [41].

## 3. Virulence

*P. aeruginosa* is well known for its high pathogenic potential, especially towards seriously ill and immunocompromised patients [43], which is related to its ability to express a wide variety of virulence factors [44]. A few of these traits also give the bacteria advantages in terms of environmental survival, allowing them to adhere to different surfaces and even form biofilms [33,45].

Some structural components of *P. aeruginosa* cells act as virulence determinants, but this species can also synthesize additional virulence factors, which can be excreted into the surrounding environment or directly into the host [44,46].

According to [47], virulence factors can be divided into three categories: cell surface structures, secreted factors, and factors involved in bacterial cell-to-cell interaction (Table 1) [44,46,47,48]. In this review, we discuss the most significant ones, especially those associated with *P. aeruginosa*’s ability to evade antimicrobial action.

### 3.1. Virulence Factors Related to the Cell Membrane

Like most Gram-negative bacteria, *P. aeruginosa* contains an outer membrane (OM) composed of lipopolysaccharide (LPS), pili, flagella, and about 300 proteins with several functions, most of which are still unknown. These structures confer advantages on this species, being involved in bacterial interaction with the host and in environmental survival [44,46].

LPS is fundamental to the integrity of the bacterial cell, but it also plays a relevant part in the host immune response and tissue damage, antibiotic resistance ability, and biofilm formation [46,49]. It consists of three domains: lipid A, core, and O-polysaccharide. Lipid A, a hydrophobic glycopeptide, is the inner portion of the LPS molecule, responsible for its endotoxicity. It promotes local and systemic inflammatory responses, necrosis, and septic shock through the disruption of cellular activities and macrophage activation [44,50]. The O-polysaccharide, or O-antigen, is the outer portion of the LPS, and is highly immunogenic, being responsible for bacterial interactions with the host or the environment, protecting it from phagocytosis and oxidative stress [49].

Motility is an important trait, as it allows bacteria to reach available nutrients. *P. aeruginosa* presents a single flagellum at one of its poles, as well as several pili and fimbriae. The flagellum is responsible for bacterial mobility, while the fimbriae allow it to adhere to host epithelial cells or environmental surfaces [44,47]. Additionally, these proteins can also initiate an inflammatory response in the host and have already been associated with drug resistance and biofilm formation ability in *P. aeruginosa* [45,49,51]. Pili play a crucial role in the initial stages of *P. aeruginosa* infections, as they contribute to bacterial twitching and swarming motility, the adhesion to surfaces, and biofilm formation [46,47].

OM proteins are involved in microbial nutrition by transporting amino acids, peptides, and carbon sources, but also antibiotics [44,52]. Additionally, they play a key role in bacterial adhesion and host recognition [44]. *P. aeruginosa* expresses twenty-six specialized channels for the transport of molecules, known as porins. These proteins can be divided into four categories, namely non-specific porins, responsible for the slow diffusion of small hydrophilic molecules; specific porins, which only allow the diffusion of particular molecules; gated porins, responsible for the uptake of ion complexes; and efflux porins, present in efflux pumps [4,12,53].

### 3.2. Secreted Virulence Factors

Most *P. aeruginosa* virulence factors are synthesized in its cytoplasm before being expelled to the extracellular space or introduced directly into the host cells through complex secretion systems, thereby avoiding elimination by the host immune system [46,54].

**Figure 1:**
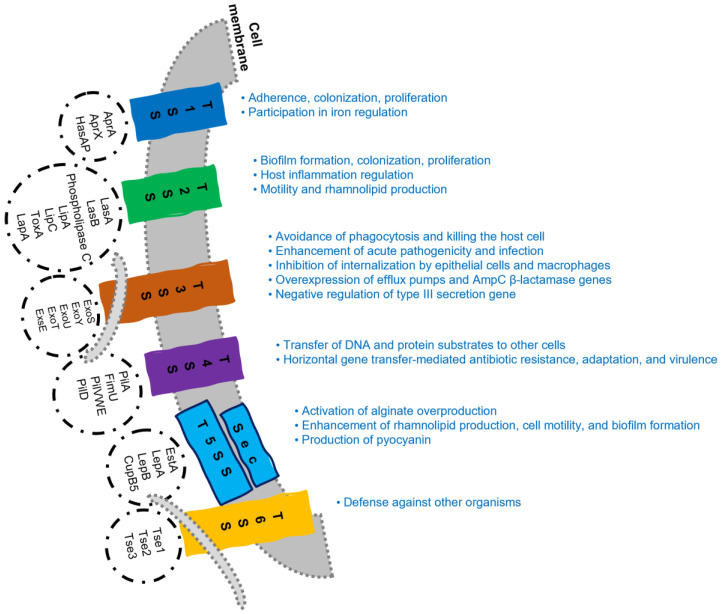
Schematic representation of *P. aeruginosa* secretion systems (T1SS to T6SS) and their functions (original).

*P. aeruginosa* expresses six types of secretion systems (Figure 1) that participate in the transport of several virulence factors, including enzymes, exotoxins, and other proteins [20,44,48,54,55,56,57,58].

These systems are divided into two groups: one-step secretion systems (T1SS, T3SS, T4SS, and T6SS) and two-step secretion systems (T2SS and T5SS), which differ in the course of protein transport. In one-step secretion systems, the protein is directly transferred from the bacterial cytosol to its surface, crossing both the inner and outer membranes in a single step. In two-step secretion systems, the protein is temporarily stored in the periplasmic space located between the inner and outer membranes, before being released to the external environment (Figure 1). These differences affect the efficiency and specificity of protein transport [20,44,55,56,57,58].

T1SS has three structural elements, an ABC transporter protein, a membrane fusion protein, and an outer membrane factor, which participate in the one-step transfer of substrates across bacterial membranes [58,59,60]. T2SS secretes folded proteins, which are first transported through the inner membrane to the periplasm via the general secretory (Sec) or twin-arginine translocation (Tat) secretion mechanisms, after which they are secreted to the cell exterior. T2SS is mainly involved in nutrient acquisition [58,59,60]. T3SS transfers virulence proteins, named effectors, from the bacterial cytoplasm into the eukaryotic cell in a one-step process [58,59,60]. T4SS, unlike other secretion systems, is capable of transferring DNA in addition to proteins [57,61]. T5SS has a Sec translocase, which allows substrates to cross the inner membrane and reach the periplasm [55,59,60]. T6SS transports toxic substrates that can act on both eukaryotic [62] and prokaryotic cells [63], playing an important role in *P. aeruginosa* pathogenesis [59,60].

Beyond secretion systems, *P. aeruginosa* produces several other virulence determinants, including enzymes such as lipases, elastase A and B, and rhamnolipids [44,46,64]. Moreover, this species is known to produce several pigments that participate in iron scavenging, cell protection, and bacterial competition [1]. Pigments produced by *P. aeruginosa* include pyocyanin, a redox-active blue pigment that causes oxidative stress in the host by interfering with electron transport [64,65]; pyoverdine, a fluorescent siderophore that helps the bacterium to acquire iron—an essential nutrient for bacterial growth and virulence—from the surrounding environment [1]; and pyorubin, a red pigment that also participates in iron acquisition and in the maintenance of the redox equilibrium of the cell [44,46].

Some other relevant substances synthesized and secreted by *P. aeruginosa* include exotoxin A, a potent cytotoxin that leads to necrosis; ExoS, that has a negative impact on the host immune system; ExoU, associated with disease severity and increased mortality [12,66,67]; and exopolysaccharides, such as alginate, Psl, and Pel, responsible for bacterial tolerance to harsh conditions, such as desiccation and the presence of oxidizing agents, and for evasion of host defenses [47,68]. They are crucial for biofilm formation as they are the main components of its matrix [68,69].

In fact, besides having an obvious role in *P. aeruginosa* pathogenicity, some of these virulence factors contribute to *P. aeruginosa*’s resilience in a variety of ecological niches, helping its communication and competition with other bacteria.

### 3.3. Quorum Sensing Systems

To regulate all these virulence factors, *P. aeruginosa* relies on a highly coordinated communication system, known as quorum sensing (QS). Due to QS, bacteria are capable of sensing variations in population density and environmental conditions and of coordinating the behavior of the bacterial population [1,70]. This communication system is based on the expression of small signaling molecules. When the concentration of these signals reaches a certain threshold, they collectively activate specific transcriptional regulators that control gene expression [44,70].

*P. aeruginosa* has four distinct QS systems: the acyl-homoserine lactone QS systems (Las and Rhl), the quinolone QS system (Pqs), and the novel QS system (Iqs). The Las system positively controls the other three. Likewise, the Iqs system positively affects Pqs, which in turn stimulates Rhl, which has a negative effect on Pqs [46,70,71,72].

In the Las system, LasI binds to the transcriptional activator LasR, regulating the transcription of different virulent factors, such as LasA, LasB, AprA, PVD, and ETA, which are involved in host cell damage and acute infections. LasI induces the production of the autoinducer N-3-oxododecanoyl-L-homoserine lactone (C12HSL), creating an autoregulatory loop [46,73]. Together with pyocyanin, C12HSL is involved in the formation of persister cells by *P. aeruginosa*, which are recognized as responsible for recalcitrant chronic infections [74]. The Las system also induces apoptosis in airway epithelial cells by destroying their tight junctions, and, along with the Rhl and Pqs systems, affects T6SS [75].

RhlI is involved in the synthesis of the autoinducer N-butyryl-L-homoserine lactone (C4HSL). C4HSL forms a complex with the activator protein RhlR, participating in biofilm formation, LecA production, and the repression of genes involved in T3SS production [46,73].

The several alkyl-4(1H)-quinolones which participate in *P. aeruginosa*’s quinolone QS system are synthesized by enzymes coded in three gene clusters (*pqsABCDE*, *phnAB*, and *pqsH*) [76]. The most common is 2-heptyl-hydroxy-1H-quinolin-4-one (PQS), which acts as a mediator in iron acquisition, cytotoxicity, and the biogenesis of outer membrane vesicles. It also stimulates neutrophil chemotaxis, prompts the production of reactive oxygen species and tumor necrosis factor-α, and impairs the secretion of cytokines IL-2 and IL-12 [76]. The Pqs system binds to the *pqsABCDE* promoter, creating a positive feedback loop and promoting PqsE production, which is the major virulence effector of the quinolone system [76]. Together with the Rhl system, PqsE is involved in the regulation of pyocyanin synthesis, the expression of genes associated with iron starvation, and the formation of efflux pumps. The Pqs system also mediates the release of extracellular DNA (eDNA), which is vital for the development of a stable and mature biofilm [46,77].

In the novel *P. aeruginosa* QS system, Iqs is driven by the signal molecule 2-(2-hydroxyphenyl)-thiazole-4-carbaldehyde (IQS) [46,73]. Iqs monitors bacterial density and detects decreases in the concentration of phosphate, an indicator of infection-associated stress, in order to regulate the production of virulence factors [65]. It also controls Las system functions and, when disrupted, pyocyanin production [65]. Importantly, IQS inhibits host cell growth and stimulates apoptosis in a dosage-dependent manner [78].

The regulation of virulence factors depends on cell density and the release of the QS autoinducers Las, Rhl, Pqs, and Iqs. Together with other virulence factors, QS systems play a major role in *P. aeruginosa* virulence and survival [46].

## 4. Antibiotic Resistance

Antibiotics are a powerful tool in the treatment of any bacterial infection. However, the emergence of pathogens resistant to multiple antimicrobial agents has become a major public health threat worldwide [79,80,81,82]. Bacteria are classified as multidrug-resistant when they are not susceptible to at least one agent in three or more classes of antibiotics; as extensively drug-resistant when they are not susceptible to at least one agent in all categories but are susceptible to two or fewer antimicrobial categories; and as pan-drug-resistant when they are not susceptible to any agents in any antimicrobial categories [83].

*P. aeruginosa* is well known for its resistance capacity towards multiple antibiotics. In fact, the most recent report published by the European Center for Disease Control states that 30.1% of the *P. aeruginosa* isolates reported are resistant to at least 1 of the antimicrobial groups under surveillance [84].

*P. aeruginosa* presents three main types of resistance mechanisms: intrinsic, acquired, and adaptative.

### 4.1. Intrinsic Resistance

Intrinsic antibiotic resistance refers to the inherent characteristics of bacteria that allow them to survive the action of antibiotics and other antimicrobial agents [6,44]. This type of resistance depends on bacterial cell structures and does not develop due to previous contact with inhibitory compounds. For instance, *P. aeruginosa* is known for its intrinsic resistance to several antibiotics, associated with the decreased permeability of its OM, the expression of efflux pump systems, and the production of antibiotic-inactivating enzymes [67,85,86]. These mechanisms may be responsible for bacterial resistance towards β-lactams and quinolones, which penetrate the cell through porin channels, and towards aminoglycosides and polymyxins, whose uptake depends on the interaction with the bacterial OM [87,88].

#### 4.1.1. OM Permeability

*P. aeruginosa* can decrease OM permeability by managing the number of non-specific porins present in the membrane [4,53]. OprF is the most prevalent porin in this bacterial species. Although non-selective, it is associated with low efficiency in antibiotic diffusion, since only a small fraction of OprF can form open channels [4,12]. OprD also participates in antibiotic uptake. It contains the binding sites for carbapenems, and low numbers of this porin confer a basal level of resistance to this class of antibiotics [89]. Additionally, OprH overexpression has been related to *P. aeruginosa*’s decreased susceptibility to polymyxin B and gentamicin, as it induces modifications in the LPS [12,90,91].

#### 4.1.2. Efflux Systems

Another mechanism associated with antibiotic resistance is the presence of efflux pumps, which remove harmful agents from the interior of the bacterial cell, helping to maintain a stable internal environment [92]. *P. aeruginosa* has active multidrug efflux pumps that contribute significantly to its antibiotic resistance ability. These pumps can be classified into five different families: the resistance nodulation division family (RND), major facilitator superfamily, ATP-binding cassette superfamily, small multidrug resistance family, and multidrug and toxic compound extrusion family [12,92,93,94].

RND is particularly involved in antibiotic resistance in *P. aeruginosa*. This bacterial species expresses four well-known active efflux pumps belonging to the RND family, including MexAB-OprM responsible for the efflux of β-lactams, including carbapenems and quinolones; MexXY/OprM involved in intrinsic resistance to aminoglycosides; MexCD-OprJ also responsible for β-lactams elimination; and MexEF-OprN responsible for quinolone extrusion [92,93,95,96,97]. The overexpression of multiple efflux pumps by this species has been associated with multidrug resistance [12,95].

#### 4.1.3. Antibiotic-Inactivating Enzymes

*P. aeruginosa* produces enzymes capable of selectively inactivating or modifying antibiotics, such as β-lactamases and aminoglycoside-modifying enzymes [98,99].

β-lactamases are hydrolytic enzymes, produced by most Gram-negative bacteria, which can inactivate β-lactam antibiotics by breaking the peptide bonds present in their molecules [100]. *P. aeruginosa* can express several β-lactamases belonging to different classes, such as penicillinases, cephalosporinases, cephamycinases, and carbapenemases, and was also found to be able to produce extended-spectrum β-lactamases, which confer resistance to an even wider range of antimicrobial agents [101,102].

*P. aeruginosa*’s resistance to aminoglycosides can be attributed to enzymatic modification that can occur through three different pathways: via aminoglycoside phosphotransferase, which can inactivate antibiotics such as neomycin and streptomycin by transferring a phosphoryl group to the 3-hydroxyl group; via aminoglycoside acetyltransferase, which inactivates gentamicin and tobramycin by transferring the acetyl group to the amino group; and via aminoglycoside nucleotidyltranferase that induce resistance to amikacin by transferring adenosine to either the amino or hydroxyl groups present in this antibiotic [103,104,105].

### 4.2. Acquired Resistance

The acquisition of new resistance determinants implies a change in the microorganism’s genetic material and can occur through gene mutation or the acquisition of new DNA by horizontal gene transfer [67,106]. Contrary to intrinsic resistance, the development of acquired resistance is highly influenced by external stressors, such as exposure to antibiotics. In the presence of such compounds, resistant bacteria present a selective advantage over susceptible strains; this way, the surviving bacteria will give rise to a resistant population [106,107,108,109].

Mutations can be induced or occur spontaneously and can be responsible for permanent changes in the bacterium gene structure. If its new traits are beneficial for the bacteria and contribute to its survival in adverse conditions, they will probably become predominant as they are transmitted to subsequent generations [110]. In *P. aeruginosa*, mutations can result in modifications of antibiotic targets or porin channels, and therefore in a reduced antibiotic uptake, or in the increased expression of resistance genes, and consequently in the overproduction of antibiotic-inactivating enzymes and multidrug efflux pumps [106,109,111].

Increased production of the AmpC enzyme is frequently observed in *P. aeruginosa*, being responsible for a high level of resistance to β-lactams, including to third- and fourth-generation cephalosporins [112,113,114]. It can occur due to mutations in the *ampC* gene itself or in the *ampD* gene, which codes for the cytosolic N-acetyl-anhydromuramil-l-alanine amidase, which is a repressor of *ampC* expression and contributes to the overproduction of β-lactamases [99,115,116].

The overexpression of efflux pump systems is another commonly acquired resistance trait in *P. aeruginosa* [117]. For example, the expression of the *mexAB-oprM* operon is negatively controlled by several regulatory loci, such as *mexR*, *nalD*, *nalB*, and *nalC*. Mutations in these loci can result in the overproduction of the MexAB-OprM efflux pump complex, leading to increased resistance to several antibiotics [92,96,117,118]. Similarly, the overexpression of MexXY-OprM, induced by mutations in *mexZ*, results in *P. aeruginosa*’s resistance to aminoglycosides, β-lactams, and fluoroquinolones [119,120,121].

Finally, mutations in genes coding for DNA gyrase (*gyrA* and *gyrB*) and topoisomerase IV (*parC* and *parE*) are associated with *P. aeruginosa*’s reduced susceptibility to quinolones. These mutations may lead to modifications of the target sites of these antibiotics, decreasing their binding affinity [122,123].

Bacteria can acquire exogenous DNA through three principal mechanisms: conjugation, in which DNA is transferred through direct cell-to-cell contact; transduction, in which bacteriophages are responsible for gene transfer between two bacterial cells; and transformation, which occurs when bacteria incorporate free DNA fragments present in the surrounding environment [12,124]. Different resistance genes can be present on plasmids, transposons, integrons, and prophages and can be transferred between bacteria belonging to the same or to different species [124,125]. For example, Liu et al. [126] have found evidence in *E. coli* of a plasmid-containing *mcr-1* gene, responsible for resistance to colistin, a last-resort antibiotic for the treatment of infections promoted by carbapenem-resistant bacteria. Additionally, the authors observed that this plasmid could be mobilized into *Klebsiella pneumoniae* and *P. aeruginosa* via conjugation [126,127]. The fact that plasmids can be transferred among unrelated Gram-negative bacteria genera is of great concern, as it allows the quick spread of resistant traits between bacterial species associated with difficult-to-treat infections [127,128].

### 4.3. Adaptative Resistance

Adaptive resistance is a mechanism used by bacteria to temporarily increase their ability to resist the effects of antibiotics or other stressors. It involves changes in gene and protein expression in response to environmental stimuli. However, this type of resistance is usually reversible when the environmental conditions become favorable [12,67].

In *P. aeruginosa*, the formation of biofilms and of persister cells are examples of those strategies. Overall, biofilm formation is influenced by the autoinducer C4HSL from Rhl QS [46,73] and by eDNA released by the Pqs system [46,77]. Moreover, LPS [46,49], flagellum and fimbriae [45,49,51], pili [46,47], the secretion systems T2SS and T5SS [44,55,58], and exopolysaccharides such as alginate, Psl, and Pel [68,69] have also been associated with biofilm formation.

Biofilms are a significant problem for both human and veterinary medicine, since biofilm-associated bacteria are protected from the action of antimicrobials and disinfectants, and also from the action of the host immune system [3]. Biofilms, the main form of bacterial growth, are ubiquitous complex structures irreversibly attached to surfaces, consisting of an interactive polymicrobial community embedded in a self-produced matrix of polymeric extracellular substances, composed by polysaccharides, proteins, and eDNA [3,33,129]. These bacterial communities are responsible for more than 50% of nosocomial infections and are recalcitrant to most antimicrobials, due to several reasons, including the incapacity of these compounds to penetrate the biofilm matrix; the sequestering of antimicrobials within the biofilm structure; the metabolic latency of the bacteria present in the inner layers of the biofilm, due to poor nutrient and oxygen availability; the establishment of communication systems between biofilm-based bacteria via autoinducer expression; the increased rate of antimicrobial resistance gene transfer between biofilm bacteria; and the presence of high numbers of persister cells [130,131]. These are a small subpopulation of cells present within a biofilm that spontaneously enter a quiescent state, displaying no signs of cell division [130]. This bacterial phenotype corresponds to metabolically inactive bacteria that have entered in a dormant state, which allows them to survive prolonged exposure to high doses of antimicrobials [132,133]. Persister formation results from changes in gene expression, being triggered by stress factors, including exposure to antimicrobials; when the antimicrobial is removed from the environment, these cells recover their metabolic activity and switch back to their antimicrobial-susceptible wildtype [130,134,135].

These phenotypes allow *P. aeruginosa* to resist disinfection treatments, contributing to its persistence and survival in the hospital setting [136]. Indeed, studies have reported the high resistance ability of *P. aeruginosa* biofilms to several biocides, including quaternary ammonium compounds, chlorine, and aldehydes [38,136,137].

Moreover, *P. aeruginosa*’s ability to survive in the hospital environment has also been associated with its ability to form biofilms that can effectively colonize inert materials, such as medical equipment and devices, including urinary catheters and implants, and that have been isolated from different types of hospital surfaces, such as faucets and water systems [9,11,28,29,30,31,32,138,139,140]. *P. aeruginosa* is qualified as an “Opportunistic Premise Plumbing Pathogen” due to its high capacity to colonize and endure within hospital water networks [141]. Hence, biofilms present in such environments can be a reservoir of opportunistic strains, including *P. aeruginosa* [142,143]. This bacterium can survive different water treatments, including sub-chlorination, application of antibiotics and biocides, and thermal shock [142,144]. *P. aeruginosa* is well adapted to the adverse conditions promoted by these treatments due to its resistance mechanisms. For example, resistance to biocides has been previously associated with low membrane permeability, efflux pump expression, horizontal gene transfer of resistance genes, bacterial genomic plasticity, and the occurrence of adaptative mutations [142,145].

The emergence of MDR and XDR clinical isolates associated with difficult-to-treat infections, such as the epidemic *P. aeruginosa* high-risk clones ST235, ST111, ST233, ST244, ST357, ST308, ST175, ST277, ST654, and ST298 [146], has already been reported in different hospitals and represents a worldwide problem [145]. According to SENTRY, *P. aeruginosa*’s resistance to piperacillin–tazobactam and meropenem between 2019 and 2021 was similar in Western Europe and the United States (23%) and higher in Eastern Europe in patients hospitalized with pneumonia (34.7%) [147].

One of the most important measures to control the dissemination of nosocomial pathogens in hospitals is to avoid hand contact with surfaces, which should be frequently disinfected [139,148].

## 5. Biocide Tolerance

Environmental contamination in healthcare facilities has been thoroughly studied and related to the transmission of nosocomial infections [149,150,151,152,153,154,155,156,157,158,159,160,161,162,163,164,165]. Therefore, cleaning and disinfection are essential for limiting pathogen spread in hospital settings, with chemical disinfectants being the preferred biocides to be applied for this purpose [166,167,168,169,170].

Biocides have a multitarget antimicrobial activity that is usually independent of the metabolic state of the bacteria, allowing them to be effective against both active and dormant cells [171,172]. However, their excessive and improper use may result in biocide tolerance or even in antimicrobial cross-resistance [173,174,175,176,177,178,179,180,181,182]. The effectiveness of biocides can be impaired by several different factors, including those related to the biocide’s chemical properties, such as its concentration, pH, and composition, and those related to the conditions in which it is applied, such as the environmental temperature, presence of organic matter, and contact time [175,176,183]. Additionally, the antibiotic resistance traits displayed by bacteria, either inherent or acquired, can also influence their susceptibility to biocides. Disinfectants differ from antibiotics in their mode of action, which is usually non-specific, and target different processes or sites within bacterial cells, rendering these chemicals very effective.

As already discussed in this review, *P. aeruginosa* is equipped with a plethora of antimicrobial tolerance mechanisms, and the overexpression of efflux pumps is frequently associated with this problem [184,185,186,187]. In fact, efflux-pump-mediated resistance towards different classes of biocides, including phenolic compounds [188,189,190,191,192,193,194], cationic biocides [185,195,196,197,198,199,200], alkylating agents [201,202,203,204], and oxidizing compounds [173,205] has already been described.

In *P. aeruginosa*, the *mexAB-oprM*, *mexCD-oprJ*, and *mexEF-oprN* genes are among the most studied regulators of efflux pumps associated with tolerance to biocides [186,193,206]. The *qac* genes, especially *qacE* and *qacEΔ1*, coded in plasmids and integrons, are frequently associated with resistance to biocides, especially to quaternary ammonium compounds, and have already been described in *P. aeruginosa*, including in both clinical and environmental strains [200,207,208,209,210,211].

The possible correlation between biocide tolerance and antibiotic resistance has been a matter of concern in the scientific community [173,184,193]. This phenomenon could result in surface disinfection failures, leading to the spread of pathogens capable of resisting both antibiotics and disinfectants used in healthcare settings. In fact, this problem can be due to co-resistance, observed when biocide tolerance and antibiotic resistance genes are located on the same mobile genetic element [212,213], or to cross-resistance, which occurs when the same mechanism is responsible for both antibiotic resistance and biocide tolerance, as observed, for example, due to the expression of efflux pumps and changes in the outer membrane permeability [213,214,215,216].

In 2010, Mc Cay et al. [217] showed that *P. aeruginosa*’s continuous exposure to subinhibitory concentrations of benzalkonium chloride resulted in its increased tolerance to this chemical agent but also contributed to a significant increase in its resistance to fluoroquinolones through mutations in *gyrA*. Tandukar et al. [218] demonstrated that such exposure is also associated with increased resistance to other clinically relevant antibiotics, such as penicillin G and tetracycline.

Overall, selective pressure due to the repeated exposure of bacteria to subinhibitory concentrations of biocides seems to play a role in the emergence of antibiotic resistance, although further research is needed to completely understand its implications in co- and cross-resistance development [171].

## 6. Conclusions

*P. aeruginosa* is a highly resilient pathogen that not only possesses a wide variety of intrinsic mechanisms of defense against external aggressions but is also able to develop new strategies to survive. It seems that the virulence factors expressed by this bacterial species contribute not only to its pathogenicity but also to its high ability to adapt to different external aggressors, such as antibiotics and biocides, and to colonize inert materials, allowing *P. aeruginosa* to thrive in the adverse conditions observed in hospital settings. This bacterium is widely known for its multi-resistant profile towards several antibiotics, including last-generation compounds, rendering it a major concern for public health.

Additionally, biocide tolerance and possibly co- and cross-resistance to antibiotics have also been reported in *P. aeruginosa*. These phenomena seem to be related to the continuous exposure to subinhibitory concentrations of biocides, which frequently occurs due to their daily use in different settings, including in healthcare, industrial, and domestic facilities. All these aspects promote a selective pressure towards highly resistant strains that will continue to prevail in the environment and consequently be transmitted to different hosts.

Therefore, it is urgent to continue investing in scientific research in this field, in order to better understand *P. aeruginosa*’s resistance mechanisms and, consequently, to develop new control strategies against this pathogen, aiming to impair its dissemination in hospital environments.

## Figures and Tables

**Table 1:** *P. aeruginosa* virulence factors.

Categories	Virulence Factors
Cell surface structures	Outer membrane (lipopolysaccharide) Appendages (type IV pili, flagella) Secretion systems (T1SS, T2SS, T3SS, T4SS, T5SS, T6SS)
Secreted factors	Exopolysaccharide (alginate, Pel, PsL) Siderophores (pyoverdine, pyochelin) Proteases (alkaline protease: AprA; elastases: Las A, LasB; protease IV) Toxins (T3SS effectors: ExoS, ExoU, ExoT, ExoY; exolysin A; exotoxin A; lipase A; phospholipase C; lipoxygenase; leukocidin; pyocyanin)
Bacterial cell-to-cell interaction	Quorum sensing Biofilm

## Data Availability

No new data were created.
